# Management and outcome of different types of ventricular tachycardia associated with hypokalemia

**DOI:** 10.1016/j.hroo.2025.05.030

**Published:** 2025-06-05

**Authors:** Micaela Ebert, Ines Masmoudi, Mandy Flechsig, Julia Mayer, Stefan Ulbrich, Leonhard Schleußner, Thomas Gaspar, Angela Zedda, Axel Linke, Sergio Richter

**Affiliations:** Division of Electrophysiology, Department of Internal Medicine and Cardiology, Heart Centre Dresden, University Hospital, Technische Universität Dresden, Dresden, Germany

**Keywords:** Ventricular tachycardia, Ventricular fibrillation, Hypokalemia, Reversible causes, Antiarrhythmic therapy, Catheter ablation

## Abstract

**Background:**

Hypokalemia is a potentially reversible cause of ventricular tachyarrhythmias (VTAs) such as polymorphic ventricular tachycardia/ventricular fibrillation (PMVT/VF) and sustained monomorphic ventricular tachycardia (SMVT). Despite its established role in arrhythmogenesis, the clinical implications of hypokalemia in patients with distinct VTA subtypes remain poorly understood.

**Objective:**

The aims of this study were to study the clinical characteristics, management, and outcome of patients with hypokalemia-associated VTAs and to investigate the prognosis of distinct VTA subtypes after correction of hypokalemia with or without additional VT–targeted therapies (VTTTs), such as catheter ablation or antiarrhythmic drugs.

**Methods:**

Consecutive patients admitted with hypokalemia-associated VTAs were analyzed after hypokalemia correction. Patients were categorized by VTA subtype and followed for VT recurrence, 24-month VT-free survival, and all-cause mortality. Those with other reversible causes of VTAs were excluded.

**Results:**

Sixty-five patients (mean age 69 ± 12 years; 20% (n = 13) women; mean left ventricular ejection fraction 32% ± 13%; 54% (n = 35) with New York Heart Association class III/IV; 8% (n = 5) with a left ventricular assist device) were studied; 68% (n = 44) presented with SMVT. Patients with SMVT were younger (65 ± 11 years vs 77 ± 10 years; *P* < .001) and had more advanced left ventricular dilation (left ventricular end-diastolic diameter 64 ± 12 mm vs 57 ± 12 mm; *P* = .03). Over a median follow-up of 18 months, 24-month VT-free survival was 28%. Patients with SMVT had higher VT recurrence (50% vs 10%; *P* = .002) and lower 24-month VT-free survival (16% vs 52%; *P* = .005) than did those with PMVT/VF. Among patients with SMVT, those receiving VTTTs (36%, (n = 16/44)) showed improved 24-month VT-free survival compared with hypokalemia correction alone (31% vs 7%; *P* = .02).

**Conclusion:**

Hypokalemia-associated VTAs are associated with advanced heart failure and linked to poor outcomes, especially in patients with SMVT. Although potassium correction may be sufficient for patients with hypokalemia-associated PMVT/VF, those with SMVT require additional VTTTs to improve outcomes.

**Key Findings**

▪ This study identifies poor overall outcomes in patients with hypokalemia-associated sustained ventricular tachyarrhythmias, even after prompt correction of hypokalemia.
▪ Patients with sustained monomorphic ventricular tachycardia (SMVT) had higher recurrence rates, higher median potassium levels, and lower ventricular tachycardia (VT)-free survival at 24 months than did those with polymorphic VT or ventricular fibrillation, who more often presented with moderate to severe hypokalemia.
▪ These findings challenge the notion that *hypokalemia-associated SMVT* is a reversible condition, instead suggesting the presence of an underlying arrhythmogenic substrate.
▪ Substrate-specific antiarrhythmic therapy significantly improves outcomes in patients with SMVT compared with potassium correction alone, highlighting the need for individualized treatment strategies.

## Introduction

Hypokalemia is the most common electrolyte disorder and is considered a potentially reversible cause of sustained ventricular tachycardia (VT) and ventricular fibrillation (VF).^1^, ^2^, ^3^ It directly contributes to ventricular arrhythmogenesis by increasing myocardial excitability, slowing conduction, and prolonging repolarization.^4^^,^^5^ Hypokalemia often exacerbates the arrhythmic risk associated with conditions such as heart failure (HF), ischemia, or certain pharmacological agents.^5^^,^^6^ Although treatment of reversible causes of ventricular tachyarrhythmias (VTAs) is thought to improve prognosis, several lines of evidence have shown similar survival rates in patients with hypokalemia-related VT/VF and those without a reversible cause of VT/VF.^7^^,^^8^ This suggests that the supposedly reversible causes may not be fully reversible or may coexist with an underlying arrhythmogenic substrate contributing to the arrhythmia mechanism.^9^^,^^10^

Despite the established link between hypokalemia and VTAs, the prevalence and prognostic impact of specific VTA subtypes, such as sustained monomorphic VT (SMVT) and polymorphic VT/VF (PMVT/VF), are not well established in this context. Furthermore, it is unclear whether correction of hypokalemia alone sufficiently reduces future arrhythmic events, as previous studies pooled sustained and nonsustained monomorphic and polymorphic VTs and VF, despite their distinct mechanisms.^3^^,^^7^^,^^11^ In particular, SMVT typically arises from reentrant circuits around myocardial fibrosis or scar, commonly associated with structural heart disease.^12^ In contrast, PMVT and VF are usually driven by multiple reentrant circuits or triggered activity associated with acute ischemia, electrolyte imbalances such as hypokalemia, QT interval prolongation, or severe bradycardia. Given the scarcity of data on the prognosis of hypokalemia-related VTAs, the recent 2022 European Society of Cardiology guidelines recommend evaluating implantable cardioverter-defibrillator (ICD) therapy on the basis of individual risk rather than VTA subtype.^8^

This study aimed to examine the clinical characteristics, management, and outcomes of patients presenting concomitantly with hypokalemia and either SMVT or PMVT/VF. Specifically, we sought to determine whether prognosis differs between these VTA subtypes after correction of hypokalemia and whether more comprehensive interventions, namely, antiarrhythmic drug (AAD) therapy and catheter ablation, are warranted and improve outcomes. By characterizing these distinct VTA presentations, our study provided insights to guide more tailored therapeutic approaches for patients with hypokalemia-related VTAs.

## Methods

### Study population

All consecutive patients who were admitted to our hospital between 2014 and 2024 because of sustained VT/VF were screened for analysis. Patients’ medical records were retrospectively reviewed for available serum potassium levels obtained from blood samples or blood gas analyses at the time of VTA presentation. Only patients with concomitant hypokalemia detected at or shortly after the time of the VTA event were considered for inclusion. Patients with other potentially reversible causes of VTA such as acute myocardial ischemia, hypoxemia, fever, or manifest hyperthyroidism were excluded from the analysis in order to minimize confounding factors that may influence the impact of hypokalemia on VTAs. Baseline clinical characteristics were collected from medical records. Specific predisposing factors such as nausea, vomiting, diarrhea, and recent (≤ 2 weeks prior to admission) increase in the dosage of non–potassium-sparing diuretics were recorded if documented in the patient’s history. The study conformed to the ethical principles of the Declaration of Helsinki and was approved by the institutional ethics committee. The requirement for informed patient consent was waived by the institutional review board because of the retrospective nature of the study and use of de-identified data.

### Acute and long-term management

All patients received oral or intravenous potassium supplementation to correct hypokalemia. In patients presenting with unstable sustained VTA, emergency external or internal defibrillation was performed. Stable sustained VTAs were terminated by external electrical cardioversion or intravenous administration of an AAD (usually amiodarone or lidocaine) after 12-lead electrocardiogram (ECG) recording of the VTA had been performed. In patients with SMVT, we assessed whether specific VT-targeted therapies (VTTTs), namely, initiation or escalation of AAD therapy or catheter ablation, were part of the management strategy. When applicable, data on type, dosage, and combination of AADs, as well as procedural data on VT ablation, were gathered.

### Definitions

*Sustained VT* was defined as VT lasting for at least 30 seconds or requiring intervention for termination.^8^ *SMVT* was defined as VT with the same QRS morphology from beat to beat and was differentiated from *PMVT*, defined as continually changing QRS morphology, and from *VF*, defined as a chaotic rhythm without identifiable distinct QRS complexes.^8^ These VTAs were identified and recorded by various techniques such as surface 12-lead ECG recording, continuous ECG monitoring, ICD recording, and external defibrillator–derived electrogram. *Electrical storm* (ES) was defined as ≥ 3 separate sustained VT/VF episodes within 24 hours before admission. *Hypokalemia* was defined as a serum potassium (K^+^) level < 3.5 mmol/l. Hypokalemia was further divided into 3 categories depending on the serum K^+^ level: mild (K^+^ level 3.0–3.5 mmol/L), moderate (K^+^ level 2.5–3.0 mmol/L), and severe (K^+^ level < 2.5 mmol/L).

### Follow-up

All patients underwent continuous in-hospital ECG monitoring after the VTA event. Outpatient follow-up was usually performed every 4–6 months and routinely included a clinical interview, 12-lead ECG recording, and ICD interrogation (if applicable). Blood sample collection and echocardiography were performed at the discretion of the treating physician. For the purpose of the study, patients had to be followed for a minimum of 6 months after the index admission. *Recurrence* was defined as any documented VT/VF episode that lasted longer than 30 seconds or was appropriately treated by the ICD. For patients followed at external institutions or who died during follow-up, data on VT recurrence and cause of death were gathered by contacting the referring hospitals or physicians.

### Study outcomes

The primary study outcome was all-cause mortality. Secondary study outcomes included VT/VF recurrence and VT/VF-free survival. Patients were divided into 2 groups for outcome analyses according to the presenting VTA subtype (SMVT vs PMVT/VF). In addition, a subgroup analysis was performed in patients with SMVT according to whether they received VTTTs or not (VTTTs+ vs VTTTs−).

### Statistical analysis

Categorical variables are reported as numbers (percentages), and numerical variables as means ± standard deviations when normally distributed or as medians with interquartile ranges (IQRs) when non-normally distributed. An unpaired *t* test was used to compare normally distributed continuous variables, and the Mann-Whitney *U* test was used to compare those that were non-normally distributed. A multivariate Cox proportional hazards analysis adjusted for age was performed to test the association between the outcome event (24-month VT-free survival) and baseline covariates. Survival curves comparing patients with SMVT vs PMVT/VF and patients with SMVT receiving VTTTs+ vs VTTTs− were estimated using the Kaplan-Meier method. A *P* value < .05 was considered statistically significant. All analyses were performed using SPSS version 29.0.1.0 (IBM Corporation, Armonk, NY).

## Results

A total of 65 patients (mean age 69 ± 12 years; 20% (n = 13) women) were included in the study. The clinical characteristics of the patient population and the 2 VTA groups are presented in Table 1. All patients had underlying structural heart disease: 40 patients (62%) had coronary artery disease, and 25 patients (38%) had nonischemic cardiomyopathy. The mean left ventricular ejection fraction was 32% ± 13%; 35 patients (54%) presented with severe HF symptoms (New York Heart Association class III/IV); and 5 patients (8%) had a left ventricular assist device implanted. The median potassium level was 2.9 (IQR 2.7–3.2) mmol/L. Thirty patients (46%) had mild, 23 (35%) moderate, and 12 (19%) severe hypokalemia (Table 2). HF medications with known effects on serum potassium levels included β-blockers in 83% (n = 54), angiotensin-converting enzyme inhibitors or angiotensin II receptor blockers in 72% (n = 47), and diuretics in 86% (n = 56) of patients. A recent increase in the dosage of non–potassium-sparing diuretics was reported in 14 patients (22%). A total of 43 patients (66%) had an ICD implanted at the time of admission for the VTA event. Thirty patients (46%) had previously experienced ≥ 1 VT/VF episode, and 13 patients (20%) had previously undergone VT ablation. AADs used included amiodarone in 16 patients (25%) and class I agents (mexiletine or flecainide) in 4 patients (6%), with amiodarone coadministered in all 3 patients on mexiletine.

**Table 1:** Baseline characteristics

Characteristics	Entire cohort (n = 65)	SMVT group (n = 44)	PMVT/VF group (n = 21)	*P*
Clinical characteristics				
Age (y)	69 ± 12	65 ± 11	77 ± 10	<.001
Men	52 (80)	38 (86)	14 (67)	.096
BMI (kg/m^2^)	29 ± 6	29 ± 6	28 ± 5	.325
Hypertension	46 (71)	30 (68)	16 (76)	.507
History of AF	40 (62)	26 (59)	14 (67)	.557
eGFR (mL/(min·1.73 m^2^))	50 (41–72)	53 (43–74)	47 (37–62)	.228
Diabetes mellitus	32 (49)	20 (46)	12 (57)	.378
COPD	7 (11)	3 (7)	4 (19)	.200
N/V/D	8 (12)	4 (9)	4 (19)	.420
Ischemic heart disease	40 (62)	26 (59)	14 (67)	.557
PAINESD risk score∗	13 ± 7	13 ± 6	15 ± 7	.227
NYHA functional class				
I	10 (15)	7 (16)	3 (14)	>.99
II	20 (31)	16 (36)	4 (19)	.157
III	27 (42)	17 (39)	10 (48)	.492
IV	8 (12)	4 (9)	4 (19)	.420
III or IV	35 (54)	21 (48)	14 (67)	.152
Echocardiographic parameters				
LVEF (%)	32 ± 13	31 ± 13	34 ± 13	.3
LVEF ≥ 50%	6 (9)	4 (9)	2 (10)	>.99
LVEF < 35%	42 (66)	30 (68)	12 (60)	.577
LVEF < 25%	19 (30)	14 (32)	5 (25)	.580
LVEDD (mm)	62 ± 12	64 ± 12	57 ± 12	.032
Electrocardiographic parameters				
QRS duration (ms)	152 ± 37	154 ± 35	148 ± 40	.520
QTc interval (ms)	492 (450–546)	480 (451–520)	515 (445–578)	.233
Medication				
β-Blocker	54 (83)	38 (86)	16 (76)	.314
Digoxin	7 (11)	2 (5)	5 (24)	.031
CCB	5 (8)	3 (7)	2 (10)	.655
Antiarrhythmic drugs	17 (26)	14 (32)	3 (14)	.133
Amiodarone	16 (25)	13 (30)	3 (14)	.182
Class I	4 (6)	4 (9)	0 (0)	.296
ACE-I/ARB	47 (72)	34 (77)	13 (62)	.195
Diuretics	56 (86)	38 (86)	18 (86)	>.99
Loop diuretics	47 (72)	33 (75)	14 (67)	.483
Thiazide diuretics	36 (55)	19 (43)	17 (81)	.004
Spironolactone	19 (29)	16 (36)	3 (14)	.067
Recent increase in diuretic dose	14 (22)	8 (18)	6 (29)	.353
VTA presentation				
Previous VT/VF episodes	30 (46)	26 (59)	4 (19)	.002
Previous VT ablation	13 (20)	12 (27)	1 (5)	.046
VT storm	28 (43)	18 (41)	10 (48)	.609
Implanted device				
None	22 (34)	12 (27)	10 (48)	.105
ICD	14 (22)	10 (23)	4 (19)	>.99
CRT-D	29 (45)	22 (50)	7 (33)	.206
LVAD	5 (8)	5 (11)	0 (0)	.166

Values are presented as mean ± SD, median (interquartile range), or n (%).

ACE-I/ARB = angiotensin-converting enzyme inhibitor/angiotensin-renin blocker; AF = atrial fibrillation; BMI = body mass index; CCB = calcium channel blocker; COPD = chronic obstructive pulmonary disease; CRT-D = cardiac resynchronization therapy–defibrillator; ICD = implantable cardioverter-defibrillator; LVAD = left ventricular assist device; LVEDD = left ventricular end-diastolic diameter; LVEF = left ventricular ejection fraction; N/V/D = nausea/vomiting/diarrhea; NYHA = New York Heart Association; PMVT/VF = polymorphic ventricular tachycardia/ventricular fibrillation; QTc = corrected QT; SMVT = sustained monomorphic ventricular tachycardia; VF = ventricular fibrillation; VT = ventricular tachycardia; VTA = ventricular tachyarrhythmia.

∗ As suggested by Santangeli and colleagues.^24^

**Table 2:** Serum potassium level at the index event

Serum potassium parameters	Entire cohort (N = 65)	SMVT group (n = 44)	PMVT/VF group (n = 21)	*P*
Serum potassium level (mmol/L)	3.0 (2.7–3.2)	3.1 (2.7–3.3)	2.8 (2.4–3.0)	.05
Severity of hypokalemia				
Mild (potassium level 3–3.5 mmol/L)	30 (46)	26 (59)	4 (19)	.002∗
Moderate (potassium level 2.5–3 mmol/L)	23 (35)	12 (27)	11 (52)	.05∗
Severe (potassium level <2.5 mmol/L)	12 (19)	6 (14)	6 (29)	.2
Moderate/severe (potassium level <3 mmol/L)	35 (54)	18 (41)	17 (81)	.003∗

Values are presented as median (interquartile range) or n (%).

PMVT/VF = polymorphic ventricular tachycardia/ventricular fibrillation; SMVT = sustained monomorphic ventricular tachycardia.

∗ Value indicate a significant *P* < .05.

### Patient characteristics and presenting VTA subtype

**Figure 1:**
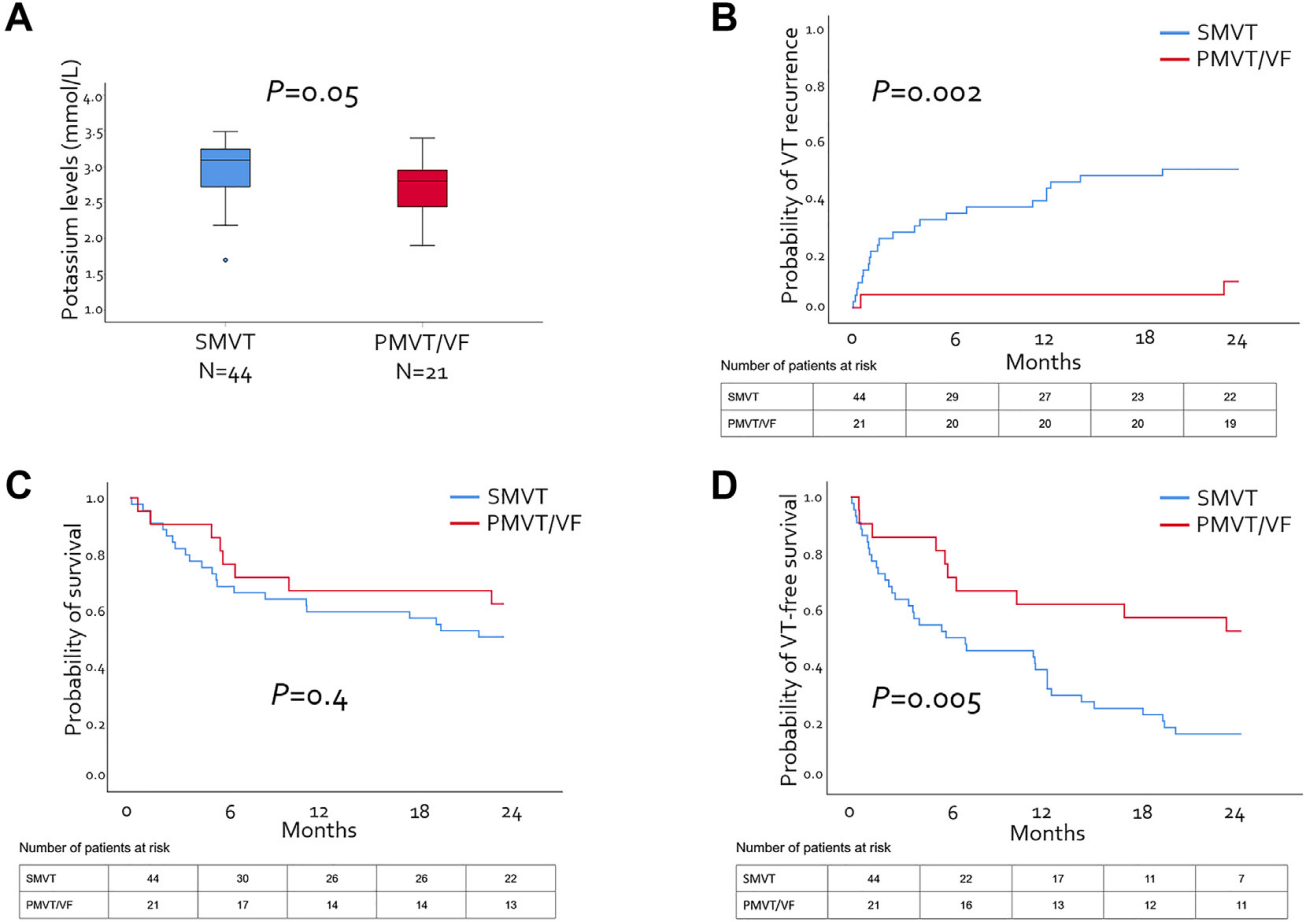
Serum potassium levels (**A**) and outcomes of patients with hypokalemia-related sustained monomorphic ventricular tachycardia (SMVT) vs polymorphic ventricular tachycardia/ventricular fibrillation (PMVT/VF) (**B–D**). **B:** Twenty-four–month ventricular tachycardia (VT) recurrence rate. **C:** Twenty-four–month survival. **D:** Twenty-four–month VT-free survival.

The documented index VTA was SMVT in 44 patients (68%) and PMVT/VF in 21 patients (32%). Twenty-eight patients (43%) presented with an ES. Patients with PMVT/VF were significantly older (77 ± 10 years vs 65 ± 11 years; *P* < .001) and more often received thiazide diuretics (81% (n = 17) vs 43% (n = 19); *P* = .004) and digitalis (24% (n = 5) vs 5% (n = 2); *P* = .031) than those with SMVT. They had lower median serum potassium levels (2.8 [IQR 2.4–3.0] mmol/L vs 3.1 [IQR 2.7–3.3] mmol/L; *P* = .05) and more often presented with significant hypokalemia (< 3.0 mmol/L) (81% (n = 17) vs 41% (n = 18); *P* = .003) (Figure 1A and Table 2). The median corrected QT interval was prolonged overall (492 [IQR 451–520] ms) and tended to be longer in patients with PMVT/VF than in those with SMVT (515 [IQR 445–578] ms vs 480 [IQR 451–520] ms; *P* = .233). There was no difference in left ventricular systolic function or renal function between the groups. Conversely, patients with SMVT had more advanced left ventricular dilation (left ventricular end-diastolic diameter 64 ± 12 mm vs 57 ± 12 mm; *P* = .032) and more often had recurrent VT/VF episodes (59% (n = 26) vs 19% (n = 4); *P* = .002) and prior VT ablation (27% (n = 12) vs 5% (n = 1); *P* = .046).

### Management of patients according to presenting VTA subtype

Correction of hypokalemia was initiated in all patients. At the time of admission for the VTA event, 22 of 65 patients (34%) were non-ICD carriers. Of these patients, 12 (55%) presented with SMVT and 10 (45%) with PMVT/VF. Four patients (18%; 3 with SMVT and 1 with PMVT/VF) were implanted with an ICD, and 5 patients (23%; 3 with SMVT and 2 with PMVT/VF) received a wearable cardioverter-defibrillator at the time of discharge. None of the wearable cardioverter-defibrillator recipients was implanted with a permanent ICD during follow-up.

Specific VTTTs were initiated significantly more often in patients with SMVT than in those with PMVT/VF (36% (n = 16) vs 5% (n = 1); *P* = .007). Catheter ablation was performed in 4 of 44 (9%) patients with SMVT and in none of the 21 patients with PMVT/VF (9% vs 0%; *P* = .29). AAD therapy was initiated or escalated in 12 of 44 patients with SMVT and 1 of 21 with PMVT/VF (27% vs 5%; *P* = .05) (Table 3). With regard to VTTTs in patients with SMVT, an AAD-based management approach was preferred over VT ablation in the majority of patients (75%, n = 12/16). This preference was associated with more severe HF symptoms (New York Heart Association class III/IV) (67% vs 25%), higher average PAINESD risk scores (13 ± 6 vs 9 ± 8), higher number of prior VT ablation procedures (3 vs 1), and higher incidence of nonischemic cardiomyopathy (67% vs 25%) in the AAD vs VT ablation group. All 5 patients with a left ventricular assist device were not considered candidates for VT ablation and were already on the maximum tolerated dose of amiodarone and/or mexiletine (Table 4).

**Table 3:** VT management

Management approach	Entire cohort (N = 65)	SMVT group (n = 44)	PMVT/VF group (n = 21)	*P*
VT-targeted therapies	17 (26)	16 (36)	1 (5)	.007∗
VT ablation	4 (6)	4 (9)	0 (0)	.3
AAD	13 (20)	12 (27)	1 (5)	.05∗
Amiodarone	10 (15)	10 (23)	0 (0)	.05∗
Sotalol	2 (3)	1 (2)	1 (5)	.6
Class I	1 (2)	1 (2)	0 (0)	1.0
De novo device management	9/22 (41)	6/12 (50)	3/10 (30)	.4
ICD/CRT-D implantation	4/22 (18)	3/12 (25)	1/10 (10)	.6
WCD	5 /22 (23)	3/12 (25)	2/10 (20)	1.0

Values are presented as n/total n (%).

AAD = antiarrhythmic drug; CRT-D = cardiac resynchronization therapy–defibrillator; ICD = implantable cardioverter-defibrillator; PMVT/VF = polymorphic ventricular tachycardia/ventricular fibrillation; SMVT = sustained monomorphic ventricular tachycardia; VT = ventricular tachyarrhythmia; WCD = wearable cardioverter-defibrillator.

∗ Values indicate a significant *P* < .05.

**Table 4:** Comparison of factors potentially driving decision making for management strategies for SMVT

Characteristics	SMVT group (n = 44)	VTTTs+ (n = 16)		VTTTs– (n = 28)
		AAD (n = 12)	VT ablation (n = 4)	
Age (y)	66 ± 11	69 ± 10		63 ± 11
		71 ± 8	62 ± 17	
eGFR (mL/(min·1.73 m^2^)	63 ± 26	63 ± 26		57 ± 22
		57 ± 18	74 ± 42	
TCL (ms)	332 ± 68	326 ± 60		336 ± 74
		330 ± 65	307 ± 40	
LVEDD (mm)	64 ± 12	60 ± 10		66 ± 12
		62 ± 9	53 ± 13	
DCM	22 (50)	9 (56)		13 (46)
		8 (67)	1 (25)	
LVAD	5 (11)	0 (0)		5 (18)
		0 (0)	0 (0)	
NYHA class III/IV	21 (48)	9 (56)		12 (43)
		8 (67)	1 (25)	
PAINESD risk score∗	13 ± 6	12 ± 7		13 ± 6
		13 ± 6	9 ± 8	
Previous VT ablation	12 (27)	4 (25)		8 (29)
		3 (25)	1 (25)	
Number of previous VT ablation procedures	1 (1)	2 (4)		1 (0)
		3 (1–5)	1 (1)	

Values are presented as mean ± SD or n (%).

AAD = antiarrhythmic drug; DCM = dilated cardiomyopathy; eGFR = estimated glomerular filtration rate; LVAD= left ventricular assist device; LVEDD = left ventricular end-diastolic diameter; NYHA = New York Heart Association; SMVT = sustained monomorphic ventricular tachycardia; TCL = tachycardia cycle length; VT = ventricular tachycardia; VTTTs+ = potassium supplementation with additional antiarrhythmic treatment; VTTTs− = potassium supplementation without additional antiarrhythmic treatment.

∗ As suggested by Santangeli and colleagues.^24^

### Follow-up and clinical outcomes

**Figure 2:**
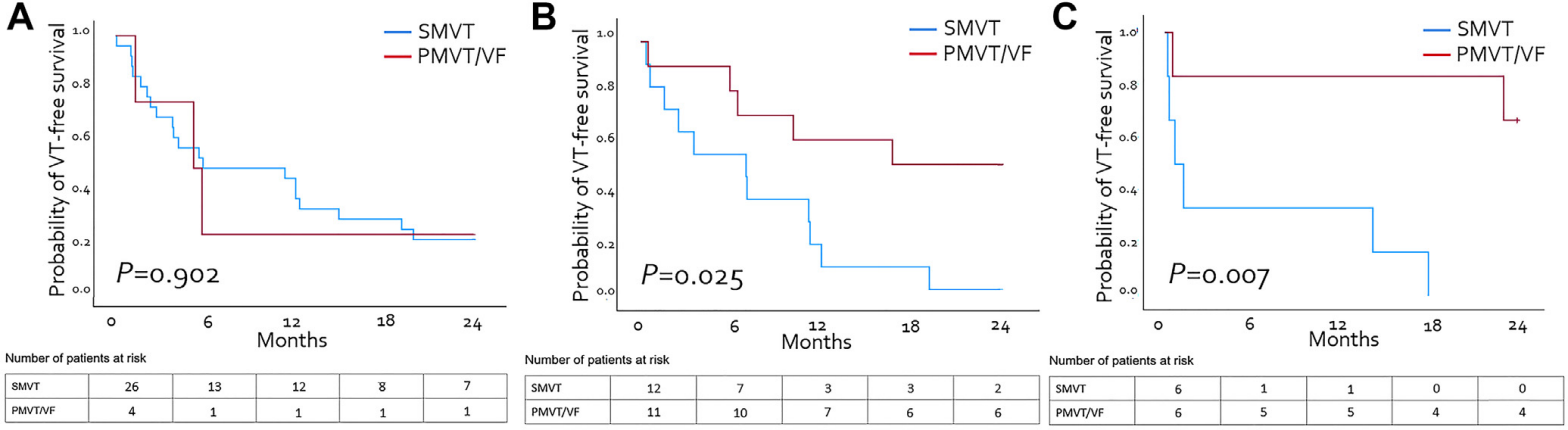
Ventricular tachycardia/ventricular fibrillation (VT/VF)–free survival according to hypokalemia severity and ventricular arrhythmia subtype. In the subgroup of patients with mild hypokalemia (**A**), there was no difference between patients with sustained monomorphic ventricular tachycardia (SMVT) and those with polymorphic VT/VF (PMVT/VF). In cases of moderate (**B**) and severe (**C**) hypokalemia, patients with PMVT/VF had significantly better VT/VF-free survival than did those with SMVT.

None of the study patients died during the index hospital stay. A total of 47 patients (72%) were discharged and followed with an implanted ICD, and 18 patients (28%) without. The median follow-up was 18 (IQR 6–30) months, with no significant difference between patients with SMVT and those with PMVT/VF (16 [IQR 5–25] months vs 24 [IQR 6–37] months; *P* = .209). Forty-one patients (63%) died during follow-up. Thirty patients (46%) experienced VT recurrence after 11 (IQR 2–24) months. The 24-month VT recurrence rate was significantly higher in patients with SMVT than in those with PMVT/VF {50% (95% confidence interval [CI] 35%–65%) vs 10% (95% CI 0%–18%); *P* = .002}. However, there was no significant difference in 24-month all-cause mortality between the groups (50% [95% CI 35%–65%] vs 38% [95% CI 17%–59%]; *P* = .353). Overall 24-month VT-free survival was 28% and significantly higher in patients with PMVT/VF than in those with SMVT (52% [95% CI 31%–74%] vs 16% [95% CI 5%–27%]; *P* = .005) (Figure 1B–1D). The presence of SMVT was the only variable that was significantly associated with impaired 24-month VT-free survival in multivariate analysis (Supplemental Table 1). Additional subgroup analyses stratified by hypokalemia severity demonstrated that patients with PMVT/VF and more severe hypokalemia had significantly better 24-month VT-free survival than did those with SMVT (Figure 2). The cause of death was known for 29 of 41 patients (71%) and was attributed to cardiac death in 14 patients (48%). Among patients with cardiac death, 11 (79%) died of end-stage HF and 3 (21%) of malignant arrhythmias (Supplemental Table 2). Eight of 41 (20%) deceased patients (5/8 (63%) with SMVT and 3 (37%) with PMVT/VF) had not undergone ICD implantation because of patient choice or palliative conditions (n = 5/8 (63%)), end-stage HF (n = 2/8, (25%)), and frailty (n = 1/8, (12%)). Two of these patients (25%, 1 with SMVT and 1 with PMVT/VF) died of unknown causes (Supplemental Table 2).

**Figure 3:**
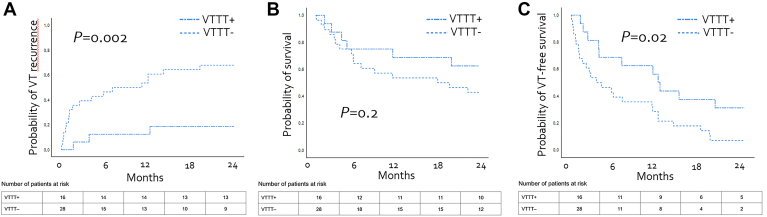
Outcomes of patients with hypokalemia-related sustained monomorphic ventricular tachycardia (SMVT) according to management strategy: ventricular tachycardia–targeted therapies (VTTTs+; potassium supplementation *with* additional antiarrhythmic treatment) vs no ventricular tachycardia–targeted therapies (VTTTs−; potassium supplementation *without* additional antiarrhythmic treatment). **A:** Twenty-four–month ventricular tachycardia (VT) recurrence rate. **B:** Twenty-four–month survival. **C:** Twenty-four–month VT-free survival.

Subgroup analyses of patients with SMVT showed significantly higher VT recurrence rates in the VTTTs− group than in the VTTTs+ group (68% [95% CI 50%–86%] vs 19% [95% CI 0%–38%]; *P* = .002). Twenty-four–month VT-free survival was also significantly lower in the VTTTs− group (2% [95% CI 0%–17%] vs 31% [95% CI 8%–55%]; *P* = .02). Patients in the VTTTs+ group had a longer median time to VT recurrence than did those in the VTTTs− group (12 [IQR 4–40] months vs 4 [IQR 1–12] months; *P* = .005) (Figure 3A and 3C). In patients with SMVT, VTTTs was the only factor associated with improved 24-month VT-free survival in univariate analysis (hazard ratio 0.362; 95% CI 0.175–0.750; *P* = .006) (Supplemental Table 3). However, there was no significant difference in 24-month all-cause mortality between VTTTs+ and VTTTs− management strategies for SMVT (38% [95% CI 13%–62%] vs 57 [95% CI 38%–67%]; *P* = .2) (Figure 3B). As presented in Supplemental Table 4, baseline clinical characteristics were similar between VTTTs+ and VTTTs– groups, suggesting that the observed difference in VT-free survival may be attributed to differences in treatment strategies rather than confounding clinical variables (Supplemental Table 4).

## Discussion

This study provides novel insights into the clinical characteristics, management, and outcomes of patients with hypokalemia-associated sustained VTA. Despite immediate correction of hypokalemia in all patients to address this potentially reversible arrhythmic cause, overall outcomes in this patient population remain notably poor. Patients with SMVT had higher VT recurrence rates and lower VT-free survival at 24 months than did those with PMVT/VF. This suggests the presence of a more complex underlying substrate in patients with SMVT, which predisposes to recurrent reentrant VT after correction of hypokalemia, thereby questioning the concept and potential reversibility of “*hypokalemia-related SMVT*.” In line with this, our study demonstrated that concomitant substrate-specific antiarrhythmic therapy improves outcomes in patients with SMVT compared with potassium correction alone.

### Prevalence, mechanisms, and characteristics of distinct VTA subtypes

Hypokalemia is a recognized trigger for VAs, with a reported prevalence ranging from 7% in real-life cohorts of unselected patients admitted to the emergency department with VT/VF to 41% in patients resuscitated from out-of-hospital cardiac arrest with defibrillator shock–treated VT/VF.^13^, ^14^, ^15^ Most of these studies focus on subsets of all-comers with VT/VF and are limited by potential confounders such as acute myocardial ischemia, with or without cardiac arrest or an ES. These conditions may lead to hypokalemia because of the underlying pathology, repeated defibrillation shocks, elevated catecholamine levels, or catecholaminergic therapy, all of which promote a transcellular shift of potassium into cells.^3^^,^^13^^,^^16^ To reduce these confounding factors and identify hypokalemia-specific contributors, patients with acute myocardial ischemia and other known transient or reversible causes for VTAs were excluded from our study.

The proarrhythmic effects of hypokalemia are attributed to the reduction in repolarization reserve resulting from the inhibition of distinct outward K^+^ currents and the outward Na^+^/K^+^-adenosine triphosphatase pump current. The latter leads to Ca^2+^ accumulation in the cytosol by the inhibition of the Na^+^/Ca^2+^ exchanger. These hypokalemia-induced changes in ionic currents result in prolonged action potential duration and increased QT dispersion and promote early and delayed afterdepolarizations. The resulting ventricular premature beats can trigger PMVT/VF but also monomorphic reentrant VT, particularly in the presence of an underlying structural substrate. Early afterdepolarization-triggered PMVT/VF occurs more commonly with hypokalemia than does SMVT.^4^^,^^5^^,^^14^ However, data on the specific prevalence of the different VTA subtypes are scarce, as they are often not differentiated or are pooled together across available studies. The lower number of PMVT/VF cases in our study may be explained by a potential selection bias due to exclusion criteria (eg, acute myocardial ischemia), which may have led to underestimation of the true prevalence of PMVT/VF. In addition, PMVT/VF more often present as a single fatal event and thus remains unrecognized in case of sudden cardiac death (SCD). In contrast, patients with SMVT may have a better chance to survive out of hospital, since they more likely present with hemodynamically stable reentrant VTs and are often equipped with an ICD for primary or secondary prevention. In line with this, patients with SMVT had more advanced left ventricular dilation, more often recurrent VT/VF episodes, and more prior VT ablation procedures than did those with PMVT/VF. These findings may serve as a surrogate for advanced left ventricular remodeling, which is often linked to structural abnormalities such as myocardial fibrosis. This suggests that SMVT in the context of hypokalemia is likely associated with a fixed arrhythmogenic substrate that predisposes to reentrant arrhythmias. In keeping with this assumption is our observation that patients with SMVT had less severe hypokalemia and shorter corrected QT intervals at the time of VTA presentation than did those with PMVT/VF, which aligns with previous studies and highlights the interplay between PMVT/VF and acute electrolyte imbalances.^14^ PMVT/VF is also associated with metabolic disturbances and medication use. In particular, thiazide diuretics and digitalis may contribute to a critical reduction in repolarization reserve and increased risk of VAs in the setting of hypokalemia. Although thiazide and loop diuretics are potentially proarrhythmogenic, thiazide diuretics are more frequently linked to PMVT/VF (especially torsades de pointes VT) because of their impact on potassium and magnesium levels.^3^^,^^17^^,^^18^ This association is reflected in our finding that significantly more patients with PMVT/VF than those with SMVT were on treatment with thiazide diuretics and digitalis.

### Clinical outcomes and prognostic considerations

Patients with VTAs attributed to transient or reversible causes, such as hypokalemia or myocardial ischemia, are often erroneously considered to have a favorable prognosis. However, our data and previous studies suggest that these patients have a similarly high risk of mortality to that of those without an identified reversible cause of VT/VF.^7^^,^^9^ This implies that the reversible factor may not be the sole trigger but could instead unmask an underlying predisposition to VAs. Therefore, these patients require a thorough evaluation, including advanced imaging to detect fibrosis, and tailored management.

Hypokalemia is a common electrolyte disturbance in patients with HF. In the setting of HF, hypokalemia often results from the interplay between the underlying heart disease, associated comorbidities, and HF medications and has frequently been associated with adverse outcomes.^11^^,^^19^, ^20^, ^21^, ^22^ This is particularly evident in the patient cohort analyzed in our study, which mostly includes individuals with advanced HF, polypharmacy and various comorbidities, such as chronic kidney disease, diabetes mellitus, and frailty. Each of these factors may not only increase the risk of hypokalemia but also independently contribute to an increased mortality.^19^^,^^21^ Thus, there is an ongoing debate about whether hypokalemia serves as an independent risk factor or primarily as a marker of the patient’s overall clinical decline, associated comorbidities, and medication use. Its direct influence on adverse and arrhythmic outcomes, as opposed to merely indicating disease severity and the effects of HF therapies, remains to be fully understood.^19^ Of note, a recent study found that in consecutive patients with any subtype of VA, 63% presented with normokalemia, 30% with hyperkalemia, and only 7% with hypokalemia.^14^ Interestingly, only hyperkalemia, but not hypokalemia, was independently associated with increased all-cause mortality after 3 years and no potassium disorder was associated with a secondary composite arrhythmic end point. These findings emphasize that other nonarrhythmic factors likely contribute to increased mortality in this population. Of note, the authors did not analyze outcomes according to distinct VTA subtype.^2^^,^^23^

Our data indicate that VTA subtype is able to predict arrhythmia-related outcomes and should therefore guide treatment decisions. Although overall 24-month VT-free survival was low (18%), patients with PMVT/VF performed significantly better than did those with SMVT (52% vs 16%). However, this composite outcome was driven by the high VT/VF recurrence rate in patients with SMVT since there was no significant difference in all-cause mortality between groups. This finding challenges the widespread assumption that solely correcting reversible causes reliably improves survival outcomes. This is reflected by the causes of death identified in our study: among patients with a documented cause of death, 52% were due to noncardiac reasons. Of the patients who died of cardiac causes, only 3 deaths were attributed to arrhythmic events while the remainder (79%) were due to end-stage HF or cardiogenic shock. Importantly, our study reflects the prognostic implications of hypokalemia in patients with structural heart disease. This limits generalizability to those without structural abnormalities, in whom the impact of hypokalemia may be more likely to be fully reversible.

### Implications for patient management according to VTA subtype

The key finding of our study is the significantly lower VT recurrence rate and longer VT-free survival in patients with SMVT who received concomitant VTTTs than in those who were managed with potassium correction alone. During 24-month follow-up, 50% of patients treated with potassium correction alone experienced VT recurrences, while this rate significantly dropped to 19% if additional interventions such as initiation or escalation of AAD therapy and/or VT ablation were performed. In contrast, potassium correction alone appeared sufficient for the acute management of patients with PMVT/VF, in whom recurrences were rare. This may be partly explained by the more severe hypokalemia observed in PMVT, potentially indicating a stronger causal link. In patients with SMVT, milder hypokalemia may reflect an underlying arrhythmogenic substrate less dependent on potassium imbalance and more likely to require extended therapy. Thus, the apparent reversibility of PMVT should be interpreted with caution, as outcomes may reflect hypokalemia severity rather than arrhythmia mechanism alone. Notably, our findings suggest that VTA subtype and hypokalemia severity may be interrelated, although causality cannot be inferred from our data, and interpretation remains limited by the small sample size. Nonetheless, these observations provide additional clinical insight into the interplay between VTA subtype and electrolyte imbalance and may help guide management strategies.

The extended VT-free survival seen in patients with SMVT who received concomitant VTTTs emphasizes the importance of addressing both the reversible metabolic trigger and the underlying arrhythmogenic substrate. Although our findings may not appear surprising from a mechanistic perspective, they provide clinically relevant real-world evidence that correction of hypokalemia alone is often insufficient in patients with SMVT. To our knowledge, this association has not yet been demonstrated in clinical studies, highlighting the novelty of our findings despite their apparent pathophysiological plausibility. These findings aim to fill a critical gap in evidence regarding antiarrhythmic management strategies in patients with hypokalemia and VTAs, providing valuable insights into treatment approaches that are underrepresented in the literature.^8^ Of note, the relatively low rate of VT ablation and preference for AADs in patients with SMVT likely reflect the severity of structural heart disease and advanced HF in our patient population. Advanced myocardial fibrosis and severe left ventricular dysfunction affect ablation efficacy and necessitate alternative, more aggressive interventions, including advanced HF therapies.

Current guidelines recommend that ICD implantation in patients with VT/VF triggered by reversible conditions should be based on individualized risk assessment for subsequent VA/SCD, without differentiation by VTA subtype.^8^ However, the high recurrence rates observed, particularly in those presenting with SMVT, challenge the notion that hypokalemia should be considered a truly reversible cause of monomorphic (reentrant) VT and that the VTA subtype may play an important role in refining future risk stratification for VTA and SCD. Notably, not all patients in our study were ICD carriers, and the absence of ICD protection in this subset may have influenced both VT recurrence and survival outcomes, particularly in cases where ICDs could have provided lifesaving arrhythmia termination. Importantly, 8 of 18 patients without an implanted ICD died, 3 of cardiogenic shock due to HF progression, 3 of noncardiac causes, and 2 of unknown causes. Although hypokalemia severity may inform clinical thinking, decisions regarding ICD implantation should not be based solely on VTA subtype or potassium levels, especially given the high risk of recurrence across subgroups. These exploratory findings require validation in larger prospective cohorts.

### Study limitations

Major limitations include the nonrandomized nature and small sample size of this single-center study. The retrospective design introduces potential selection bias, especially with regard to eligibility for VT ablation, which was limited to patients with less severe structural heart disease. Furthermore, our study lacks data on serum magnesium levels and supplemental magnesium administration. Although hypomagnesemia is known to play a role in arrhythmogenesis, the *Electrolyte Abnormalities in Patients Presenting With Ventricular Arrhythmia* study, which examined patients with VT/VF and concomitant hypokalemia, revealed a low prevalence of hypomagnesemia and no significant differences in average magnesium levels compared to patients with HF without VA.^3^ This suggests that low magnesium levels may not be a primary driver of VT/VF in this patient population. However, future studies are needed to assess the role of magnesium and benefit of its supplemental administration in hypokalemia-related VAs. In addition, potassium levels at the time of arrhythmia recurrence were not consistently documented, limiting our ability to determine whether recurrences were related to hypokalemia. This restricts causal inference regarding the protective effect of potassium correction.

## Conclusion

Our study highlights the poor prognosis of hypokalemia-associated VTAs and emphasizes the importance of distinguishing between VTA subtypes in patients with hypokalemia for management strategies. Although potassium correction is crucial, patients with SMVT often require additional antiarrhythmic interventions to improve outcomes. Although hypokalemia may not be a primary driver of SMVT in structural heart disease, its presence may reflect a more severe arrhythmogenic substrate. These findings support a more individualized approach, particularly in SMVT, and warrant further investigation to refine treatment strategies.
