# Supplementary material for: Management and outcome of different types of ventricular tachycardia associated with hypokalemia
Source: Heart Rhythm O2. 2025 Jun 5;6(9):1391–400. doi: 10.1016/j.hroo.2025.05.030 (PMC12635743; doi:10.1016/j.hroo.2025.05.030)
Supplement: Supplemental Tables [file mmc1.docx]

Supplementary data

|  | **Univariate Analysis** | | | **Multivariate Analysis** | | |
| --- | --- | --- | --- | --- | --- | --- |
|  | **HR** | **95% CI** | **p-value** | **HR** | **95% CI** | **p-value** |
| Age | 0.982 | 0.957 – 1.008 | 0.169 | 0.991 | 0.964 – 1.019 | 0.534 |
| Male gender | 1.859 | 0.831 – 4.160 | 0.131 | – | – | – |
| SMVT | 2.078 | 1.029 – 4.194 | **0.041** | 1.919 | 0.910 – 4.046 | 0.087** |
| Serum K^+^ level <3 mmol/L | 0.816 | 0.459 – 1.448 | 0.487 |  |  |  |
| BMI | 0.991 | 0.932 – 1.053 | 0.771 | – | – | – |
| PAINESD Risk Score^#^ | 1.001 | 0.957 – 1.048 | 0.956 | – | – | – |
| LVEF ≤35% | 1.036 | 0.571 – 1.881 | 0.907 | – | – | – |
| LVEDD ≥60mm | 0.726 | 0.601 – 2.075 | 0.726 | – | – | – |
| QRS | 1.007 | 0.998 – 1.016 | 0.107 | – | – | – |
| QT | 1.001 | 0.998 – 1.004 | 0.379 | – | – | – |
| IHD | 0.848 | 0.473 – 1.519 | 0.580 | – | – | – |
| DCM/NICM | 1.034 | 0.581 – 1.838 | 0.911 | – | – | – |
| Hypertension | 1.354 | 0.702 – 2.611 | 0.366 | – | – | – |
| AF | 1.040 | 0.577 – 1.873 | 0.897 | – | – | – |
| CKD |  |  |  |  |  |  |
| VT-storm | 0.876 | 0.532 – 1.712 | 0.876 | – | – | – |
| NYHA III/IV | 1.009 | 0.569 – 1.792 | 0.975 | – | – | – |

**Supplementary Table 1**. Cox Proportional Hazards Analysis of Baseline Covariates in Relation to 24-month VT-free Survival. ^#^as suggested by Santangeli et al.^24^; AF: atrial fibrillation, BMI: body mass index, CKD: Chronic kidney disease (eGFR <60ml/min), HTN: hypertension, IHD: ischemic heart disease, K^+^: Potassium, LVEF: left ventricular ejection fraction, LVEDD: left ventricular enddiastolic diameter, MI: myocardial infarction, NYHA: New York Heart Association, SMVT: sustained monomorphic VT, TCL: tachycardia cycle length, VT: ventricular tachycardia

|  | **Entire cohort**  **(n=65)** | | **SMVT group**  **(n=44)** | | **PMVT/VF group**  **(n=21)** | |
| --- | --- | --- | --- | --- | --- | --- |
|  | **ICD**  **(n=47)** | **No ICD**  **(n=18)** | **ICD**  **(n=35)** | **No ICD (n=9)** | **ICD**  **(n=12)** | **No ICD**  **(n=9)** |
| **Death, *n (%)*** | **41 (63)** | | **28 (62)** | | **13 (62)** | |
|  | 33/41 (80) | 8/41 (20) | 23/28 (82) | 5/28 (18) | 10/13 (77) | 3/13 (23) |
| **Known cause of death**, *n (%)* | **29/41 (71)** | | **18/28 (64)** | | **11/13 (85)** | |
|  | 23/29 (79) | 6/29 (21) | 14/18 (78) | 4/18 (22) | 9/11 (82) | 2/11 (18) |
| Cardiac death | 14/29 (48) | | 10/18 (56) | | 4/11 (36) | |
|  | 11/14 (79) | 3/14 (21) | 7/14 (50) | 3/4 (75) | 4/4 (100) | 0 |
| Incessant VT or electrical storm | 3/29 (10) | | 3/18 (17) | | 0/11 (0) | |
|  | 3/3 (100) | 0 | 3/3 (100) | 0 | 0 | 0 |
| Cardiogenic shock/end-stage heart failure | 11/29 (38) | | 7/18 (39) | | 4/11 (36) | |
|  | 8/11 (73) | 3 | 4/7 (57) | 3/7 (43) | 4/4 (100) | 0 |
| Other (non-cardiac)* | 15/29 (52) | | 8/18 (44) | | 7/11 (64) | |
|  | 12/15 (80) | 3/15 (20) | 7/8 (88) | 1/8 (12) | 5/7 (71) | 2/7 (29) |
| **Unknown cause of death**, *n (%)* | **12/41 (29)** | | **10/28 (36)** | | **2/13 (15)** | |
|  | 10/12 (83) | 2/12 (17) | 9/10 (90) | 1/10 (10) | 1/2 (50) | 1/2 (50) |

**Supplementary Table 2. Causes of Death and distribution of ICD carriers.** *including stroke, septic shock, respiratory insufficiency, renal failure, cancer

|  | **Univariate Analysis** | | | **Multivariate Analysis** | | |
| --- | --- | --- | --- | --- | --- | --- |
|  | HR | 95% CI | *P*-value | HR | 95% CI | *P*-value |
| Age | 1.000 | 0.967 – 1.034 | 1.000 | – | – | – |
| Male gender | 0.938 | 0.364 – 2.418 | 0.895 | – | – | – |
| VTTT+ | 0.362 | 0.175 – 0.750 | **0.006** | – | – | – |
| Serum K^+^ level <3 mmol/L | 1.720 | 0.895 – 3.306 | 0.104 | – | – | – |
| TCL | 1.005 | 0.999 – 1.010 | 0.103 | – | – | – |
| BMI | 0.987 | 0.920 – 1.058 | 0.703 | – | – | – |
| PAINESD Risk Score^#^ | 1.003 | 0.948 – 1.060 | 0.930 | – | – | – |
| LVEF ≤35% | 0.768 | 0.388 – 1.517 | 0.447 | – | – | – |
| LVEDD ≥60 mm | 0.805 | 0.398 – 1.629 | 0.547 | – | – | – |
| QRS | 1.008 | 0.998 – 1.018 | 0.126 | – | – | – |
| QTc | 1.002 | 0.997 – 1.006 | 0.457 | – | – | – |
| IHD | 1.055 | 0.539 – 2.063 | 0.876 | – | – | – |
| Previous MI | 1.797 | 0.838 – 3.855 | 0.132 | – | – | – |
| HTN | 1.547 | 0.725 – 3.298 | 0.259 | – | – | – |
| AF | 1.053 | 0.538 – 2.060 | 0.881 | – | – | – |
| CKD | 1.277 | 0.531 – 3.071 | 0.585 | – | – | – |
| VT-storm | 0.885 | 0.447 – 1.752 | 0.726 | – | – | – |
| NYHA III-IV | 0.955 | 0.498 – 1.828 | 0.889 | – | – | – |

**Supplementary Table 3.** Cox Proportional Hazards Analysis of Baseline Covariates in Relation to 24-month VT-free Survival among SMVT patients. ^#^as suggested by Santangeli et al.^24^

AF: atrial fibrillation, BMI: body mass index, CKD: Chronic kidney disease (eGFR <60ml/min), HTN: hypertension, IHD: ischemic heart disease, K^+^: Potassium, LVEF: left ventricular ejection fraction, LVEDD: left ventricular enddiastolic diameter, MI: myocardial infarction, NYHA: New York Heart Association, TCL: tachycardia cycle length, VT: ventricular tachycardia, VTTT+, potassium supplementation plus additional antiarrhythmic treatment

|  | **SMVT group**  **(n=44)** | **VTTT+ group**  **(n=16)** | **VTTT- group**  **(n=28)** | **p-value*** |
| --- | --- | --- | --- | --- |
| **Clinical characteristics** |  |  |  |  |
| Age, *years* | 65 ± 11 | 69 ± 10 | 63 ± 11 | 0.123 |
| Male, *n (%)* | 38 (86) | 12 (75) | 26 (93) | 0.097 |
| BMI | 29 ± 6 | 27 ± 5 | 30 ± 6 | 0.136 |
| Hypertension, *n (%)* | 30 (68) | 9 (56) | 21 (75) | 0.199 |
| History of AF, *n (%)* | 26 (59) | 9 (56) | 17 (61) | 0.772 |
| eGFR, *ml/min/1,73m²* | 63 ± 26 | 63 ± 23 | 57 ± 22 | 0.353 |
| Diabetes mellitus, *n (%)* | 20 (46) | 7 (44) | 13 (46) | 0.864 |
| COPD, *n (%)* | 3 (7) | 0 (0) | 3 (11) | 0.175 |
| N/V/D, *n (%)* | 4 (9) | 1 (6) | 3 (11) | 0.620 |
| Ischemic heart disease, *n (%)* | 26 (59) | 10 (63) | 16 (57) | 0.728 |
| PAINESD Risk Score^#^ | 13 ± 6 | 12 ± 7 | 13 ± 6 | 0.672 |
| NYHA functional class, *n (%)* |  |  |  |  |
| III or IV | 21 (48) | 9 (56) | 12 (43) | 0.392 |
| **Echocardiographic parameters** |  |  |  |  |
| LVEF, % | 31 ± 13 | 33 ± 14 | 29 ± 12 | 0.965 |
| LVEDD, mm | 64 ± 12 | 60 ± 10 | 66 ± 12 | 0.194 |
| **Electrocardiographic parameters** |  |  |  |  |
| QRS, ms | 154 ± 35 | 153 ± 34 | 155 ± 37 | 0.095 |
| QTc, ms | 469 ± 105 | 471 ± 114 | 494 ± 72 | 0.262 |
| **Medication, *n (%)*** |  |  |  |  |
| ß-blocker | 38 (86) | 14 (88) | 24 (86) | 0.868 |
| Digoxin | 2 (5) | 1 (6) | 1 (4) | 0.682 |
| CCB | 3 (7) | 2 (13) | 1 (4) | 0.910 |
| Antiarrhythmic drugs | 14 (32) | 11 (69) | 3 (11) | 0.159 |
| Amiodarone | 13 (30) | 2 (13) | 11 (39) | 0.061 |
| Class I | 4 (9) | 1 (6) | 3 (11) | 0.620 |
| ACE-I/ARB | 34 (77) | 13 (81) | 21 (75) | 0.634 |
| Diuretics | 38 (86) | 13 (81) | 25 (89) | 0.455 |
| Loop diuretics | 33 (75) | 12 (75) | 21 (75) | 1.000 |
| Thiazide diuretics | 19 (43) | 5 (31) | 14 (50) | 0.227 |
| Spironolactone | 16 (36) | 5 (31) | 11 (39) | 0.594 |
| Recent increase in diuretic dose | 8 (18) | 2 (13) | 6 (21) | 0.460 |
| **VTA presentation** |  |  |  |  |
| TCL, ms | 332 ± 68 | 326 ± 60 | 336 ± 74 | 0.731 |
| Previous VT/VF episodes, *n* (%) | 26 (59) |  |  |  |
| Previous VT ablation, *n (%)* | 12 (27) | 4 (25) | 8 (29) | 0.798 |
| VT-storm, *n (%)* | 18 (41) | 7 (44) | 11 (39) | 0.772 |
| **Implanted device, *n (%)*** |  |  |  |  |
| None | 12 (27) | 5 (31) | 7 (25) | 0.100 |
| ICD | 10 (23) | 2 (13) | 8 (29) | 0.221 |
| CRT-D | 22 (50) | 9 (56) | 13 (46) | 0.531 |
| LVAD | 5 (11) | 0 (0) | 5 (18) | 0.073 |

Supplementary Table 4. Clinical characteristics of patients with sustained monomorphic ventricular tachycardia with and without additional antiarrhythmic treatment (VTTT+ vs. VTTT-). ^#^as suggested by Santangeli et al.^24^ ACE-I/ARB: angiotensin-converting-enzyme-inhibitor/angiotensin-renin-blocker AF: atrial fibrillation, BMI: body mass index, CCB: calcium channel blocker, CKD: Chronic kidney disease (eGFR <60ml/min), COPD: chronic obstructive pulmonary disease, CRT-D: cardiac resynchronization therapy-defibrillator, ICD: implantable cardioverter defibrillatore, LVEF: left ventricular ejection fraction, LVEDD: left ventricular enddiastolic diameter, LVAD: left ventricular assist device N/V/D: nausea/vomiting/diarrhea, NYHA: New York Heart Association, PMVT/VF: polymorphic ventricular tachycardia/ventricular fibrillation, SMVT: sustained monomorphic VT, TCL: tachycardia cycle length, VTA: ventricular tachyarrhythmia VT: ventricular tachycardia
